# Anemia and hemoglobin thresholds of 12.5 and 13.0 g/dL are associated with distinct cardiovascular and all-cause mortality risks in COPD

**DOI:** 10.1016/j.ijcrp.2026.200691

**Published:** 2026-08-07

**Authors:** Yan Wang, Yushan Shi

**Affiliations:** a Department of Laboratory Medicine, Affiliated Hospital of Shandong University of Traditional Chinese Medicine, Jinan, Shandong, China; b Shandong University of Traditional Chinese Medicine, Jinan, Shandong, China; c Respiratory and Critical Medicine, Affiliated Hospital of Shandong University of Traditional Chinese Medicine, Jinan, Shandong, China

**Keywords:** Anemia, Hemoglobin level, COPD, Mortality, Cohort study, Propensity score

## Abstract

**Background:**

Anemia is a prevalent comorbidity in chronic obstructive pulmonary disease (COPD), yet prior studies have predominantly focused on all-cause mortality, leaving the non-linear relationship between hemoglobin and mortality—and clinically actionable thresholds—undefined. We aimed to identify distinct hemoglobin thresholds associated with cardiovascular and all-cause mortality and evaluate hemoglobin as a prognostic risk indicator in COPD.

**Methods:**

This prospective cohort study utilized data from seven NHANES cycles (2005–2018), including 1338 U S. adults with COPD representing approximately 7.7 million non-institutionalized COPD patients. Weighted Cox proportional hazards models and restricted cubic spline analysis were conducted to explore the association between hemoglobin level and mortality in COPD.

**Results:**

The weighted prevalence of anemia among U.S. adults with COPD was 10.5%. Anemia was significantly associated with all-cause mortality (HR = 2.32, 95% CI: 1.85–2.92) and cardiovascular mortality (HR = 3.75, 95% CI: 2.06–6.83). Each 1 g/dL hemoglobin increment was associated with 17% and 22% risk reductions, respectively. RCS analysis identified nonlinear relationships with thresholds at 12.5 g/dL for all-cause and 13.0 g/dL for cardiovascular mortality. Findings remained robust across all sensitivity analyses.

**Conclusions:**

Anemia and lower hemoglobin levels were independently associated with significantly increased risks of all-cause and cardiovascular mortality. Hemoglobin thresholds of 12.5 g/dL and 13.0 g/dL were identified for all-cause and cardiovascular mortality, respectively, as prognostic indicators warranting further validation. These findings underscore the importance of hemoglobin assessment in risk stratification; however, interventional studies are needed to determine whether correction of anemia improves survival in this population.

## Highlights

• Both anemia status and continuous hemoglobin levels were independently associated with all-cause and cardiovascular mortality in COPD, with cardiovascular outcomes demonstrating substantially stronger associations (HR 3.75 vs. 2.32).
• Non-linear dose-response relationships revealed distinct hemoglobin thresholds of 12.5 g/dL for all-cause mortality and 13.0 g/dL for cardiovascular mortality, below which mortality risk increased significantly.
• Findings remained robust across seven propensity score approaches (matching, adjustment, IPTW, SMRW, PA, and OW), supporting the integration of hemoglobin assessment into routine COPD risk stratification as a modifiable clinical indicator.

## 1 Introduction

Chronic obstructive pulmonary disease (COPD) is an inflammatory disorder of the lungs that affects about 10% of the adult population, and it represents the third leading cause of mortality worldwide [1,2]. COPD is characterized by persistent airflow limitation and progressive respiratory symptoms. This condition imposes a substantial and growing global health burden, contributing to significant morbidity, functional impairment, and premature mortality [3,4]. Beyond the pulmonary manifestations, COPD is increasingly recognized as a complex systemic disorder characterized by multisystem comorbidities—including cardiovascular disease (CVD), metabolic dysfunction, renal impairment, psychiatric and skeletal muscle wasting—which materially influence disease trajectory, prognosis, and healthcare utilization [5,6]. Among these comorbidities, CVD represents the leading cause of death in COPD populations, accounting for approximately 25-30% of all deaths [6]. Systemic inflammation, oxidative stress, and chronic hypoxemia—hallmarks of COPD—create a milieu that simultaneously promotes atherosclerosis, myocardial dysfunction, and erythropoietic dysregulation, making anemia a potentially informative yet underrecognized cardiovascular risk marker in this population [[7], [8], [9]]. Early recognition and targeted intervention for comorbidities are therefore warranted.

Anemia, defined by reduced hemoglobin concentration or hematocrit, represents a common hematologic complication of chronic inflammatory conditions and chronic diseases [10,11]. Clinically, anemia has been consistently associated with fatigue, reduced exercise tolerance, impaired quality of life, and worse clinical outcomes across a spectrum of chronic illnesses, including heart failure, chronic kidney disease, and malignancy [7,12,13]. Emerging yet heterogeneous evidence indicates that anemia is more prevalent in COPD than in the general population. It may portend adverse outcomes, including increased exacerbation frequency and higher mortality rates [14,15]. Mechanistically, anemia in COPD may arise from multiple interacting pathways: chronic inflammation-driven iron restriction and impaired erythropoietin response, nutritional deficiencies, renal impairment, and the myelosuppressive effects of chronic hypoxemia [16,17]. Importantly, anemia may also exacerbate cardiovascular risk in COPD through reduced oxygen-carrying capacity, which compounds tissue hypoxia, increases myocardial workload, and promotes adverse ventricular remodeling [7]. However, existing studies are limited by modest sample sizes, incomplete adjustment for key confounders (such as chronic kidney disease [CKD] and cardiovascular comorbidity), and sparse data on cause-specific mortality—particularly cardiovascular mortality [6,8].

Critically, prior investigations have predominantly examined all-cause mortality as the primary endpoint, with limited attention to cardiovascular-specific outcomes despite CVD being the leading cause of death in COPD populations [14,15,18] Furthermore, the relationship between hemoglobin and mortality has been largely modeled as linear, potentially obscuring clinically meaningful thresholds where risk acceleration occurs. Whether distinct hemoglobin thresholds exist for cardiovascular versus all-cause mortality—and whether these thresholds differ from current anemia diagnostic criteria—remains unknown.

Given these gaps, large population-representative analyses with robust mortality linkage are needed. Such studies should evaluate dose-response and threshold effects to clarify whether anemia independently predicts all-cause and cardiovascular mortality in COPD. These findings may inform risk stratification and generate hypotheses for future interventional research. This study leverages nationally representative NHANES data with linked mortality data to examine associations between anemia, hemoglobin concentration, and both all-cause and cardiovascular mortality in adults with COPD.

## 2 Methods

### 2.1 Data resource

This population-based cohort study utilized data from the National Health and Nutrition Examination Survey (NHANES) database. NHANES is conducted by the Centers for Disease Control and Prevention (CDC) to assess the health and nutritional status of the non-institutionalized U.S. population. Data were collected through a complex multi-stage stratified probability sampling method to ensure national representativeness. Comprehensive information regarding the survey is available at https://www.cdc.gov/nchs/nhanes/index.htm. NHANES protocol has been approved by the CDC National Center for Health Statistics Institutional Review Board, and all participants provided written informed consent.

### 2.2 Study participants

**Fig. 1:**
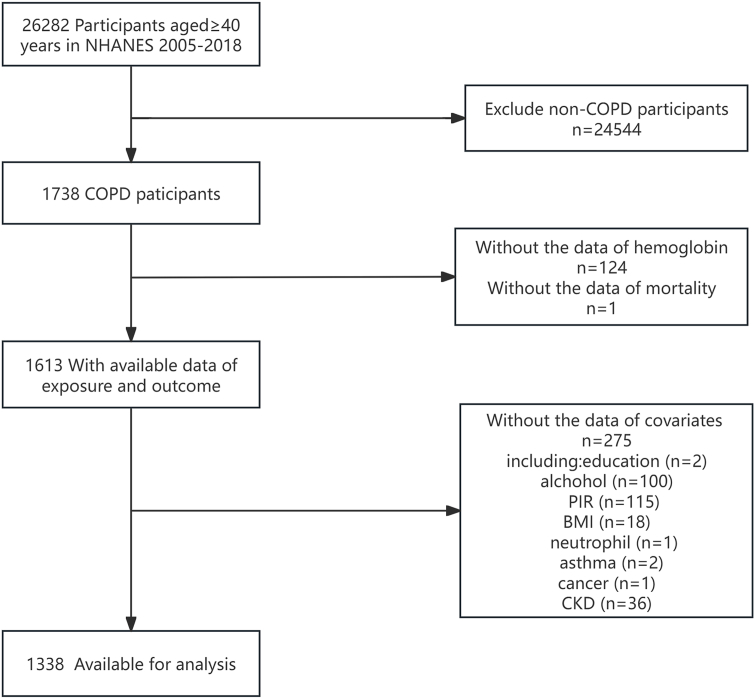
Flow diagram of the screening and enrollment of study participants. **Abbreviation:** NHANES, National Health and Nutrition Examination Survey; COPD, chronic obstructive pulmonary disease; PIR, poverty income ratio; BMI, body mass index; CKD, chronic kidney disease.

Initially, 26,282 participants aged ≥ 40 years were recruited from NHANES (2005-2018 years). Subsequently, 1738 individuals with COPD were identified. Participants with missing mortality data (n = 1), hemoglobin data (n = 124), or important covariates (n = 275) were excluded. Missing data for specific covariates were as follows: education level (n = 2), drinking status (n = 100), poverty income ratio (n = 115), body mass index (n = 18), neutrophil count (n = 1), asthma history (n = 2), CKD (n = 36), and cancer history (n = 1). The final analysis included 1338 eligible participants. The participant selection process is illustrated in Fig. 1.

### 2.3 Assessment of anemia

According to WHO standards, anemia is defined as a hemoglobin level below 12 g/dL in females and below 13 g/dL in males [19,20]. Hemoglobin was measured using the Beckman Coulter method. All laboratory assessments were conducted at Mobile Examination Centers.

### 2.4 Definitions of COPD

COPD diagnosis was determined based on one or more of the following criteria, consistent with prior NHANES-based studies using composite case definitions to maximize sensitivity [[21], [22], [23]]: post-bronchodilator FEV_1_/FVC ratio <0.70; (2) physician-confirmed diagnosis of emphysema or COPD; or (3) for participants aged ≥40 years, a history of smoking combined with chronic bronchitis and current treatment with phosphodiesterase-4 inhibitors, mast cell stabilizers, leukotriene modifiers, or inhaled corticosteroids.

### 2.5 Mortality data

Mortality data were obtained by linking the cohort database with the National Death Index (NDI). All-cause mortality was defined as death from any cause. Cardiovascular mortality was defined using International Classification of Diseases, Tenth Revision (ICD-10) codes I00-I09, I11, I13, I20-I51, and I60-I69 [24,25]. All participants were followed until death or the end of the study period (December 31, 2019).

### 2.6 Covariates assessment

Confounding variables were selected based on clinical significance and prior research [15,26,27], These included age, sex, race, marital status, educational level, poverty income ratio (PIR), body mass index (BMI), smoking status, drinking status, physical activity duration, lymphocyte count, platelet count, neutrophil count, and histories of cardiovascular disease (CVD), hypertension, diabetes, chronic kidney disease (CKD), asthma, and cancer. Racial: non-Hispanic White, non-Hispanic Black, Mexican American, other Hispanic types, and others. Marital status: married/living with partner, never married/other. Educational level: less than high school, high school and above. Economic status was classified by PIR as <1.30, 1.30–3.49, or ≥3.50 [28]. Obesity was defined as BMI >30 kg/m^2^, calculated from measured height and weight. Smoking status: never (less than 100 cigarettes in lifetime), former (more than 100 cigarettes but quit), and current (more than 100 cigarettes and currently smoking) [29]. Alcohol consumption was categorized as never, former, or current [21]. Physical activity duration was assessed via self-reported weekly duration of walking, cycling, household chores, occupational activity, and recreational activities. CKD was defined as estimated glomerular filtration rate (eGFR) < 60 mL/min/1.73 m^2^, albuminuria ≥30 mg/g, or both [30]. Diagnoses of CVD, hypertension, diabetes, asthma and cancer were self-reported. CVD including congestive heart failure (MCQ160b), coronary heart disease (MCQ160c), angina (MCQ160d), myocardial infarction (MCQ160e), or stroke (MCQ160f), consistent with established definitions in prior NHANES-based studies [31].

### 2.7 Statistical analysis

Continuous variables are expressed as mean (standard error [SE]), and categorical variables as unweighted frequencies or weighted percentages. Between-group differences were assessed using chi-square tests and t-tests. All analyses incorporated sampling weights to account for the complex multistage design of NHANES.

To assess the association between hemoglobin level and anemia and mortality in COPD participants, this study constructed four survey-weighted cox regression models. Crude model: not adjusted; model 1: adjusted for age, sex, race, marital status, education level, smoking status, drinking status; model 2: further adjusted for physical activity time, PIR, BMI, lymphocyte cell counts, PLT, neutrophils cell counts; model 3: further adjusted for hypertension, diabetes, CVD, CKD, asthma, cancer.

Nonlinear associations between hemoglobin levels and mortality were examined using restricted cubic spline (RCS) regression. Knots were placed at the 5th, 35th, 65th, and 95th percentiles to capture potential non-linearity across the distribution while minimizing overfitting. Nonlinearity was evaluated using the likelihood ratio test.

Kaplan-Meier survival analysis was performed to examine the association between anemia and mortality. Survival distributions were compared using the log-rank test.

Stratified analyses were conducted by age (<65 or ≥65 years), sex (female or male), BMI (<30 or ≥30 kg/m^2^), CVD status (yes or no), and asthma status (yes or no) to evaluate potential effect modification. Interaction effects were assessed by including multiplicative interaction terms in the models.

Comprehensive sensitivity analyses were performed to validate the robustness of findings. First, to minimize reverse causation, participants who died within two years of baseline were excluded. Second, unweighted Cox regression was performed. Third, complete case analysis was used as the primary approach, with multiple imputation performed as sensitivity analysis. Multiple imputation by chained equations was used to handle missing covariates, and analyses were repeated across five imputed datasets. To address potential confounding by indication, propensity score analyses were performed. Propensity scores were estimated using logistic regression including all covariates from model 3. Seven propensity score methods were applied: (1) multivariable-adjusted Cox regression (reference); (2) propensity score adjustment; (3) 1:1 propensity score matching (caliper width, 0.1); (4) inverse probability weighting (IPTW); (5) standardized mortality ratio weighting (SMRW); (6) pairwise algorithmic weighting (PA); and (7) overlap weighting (OW). Statistical analyses were performed using R software (version 4.2.2; R Foundation for Statistical Computing, Vienna, Austria) and Free Statistics software (version 1.9.2). A two-sided P value < 0.05 was considered statistically significant.

## 3 Results

### 3.1 Baseline characteristics of participants

1338 participants with available data for analysis are shown in Table 1, representing approximately 7.70 million US COPD adults aged ≥40 years. The weighted prevalence of anemia in this COPD population was 10.5%. The mean (SE) age was 61.85 (0.34) years. Of the participants, 50.9% were male, and the mean (SE) hemoglobin concentration was 14.27 (0.07) g/dL. Compared with participants without anemia, those with anemia were significantly older (mean age 66.10 (1.12) years vs. 61.36 (0.35) years, p < 0.001). The anemia group exhibited significantly higher prevalence of cardiovascular disease, hypertension, diabetes, and chronic kidney disease. Significant differences in all-cause and cardiovascular mortality were observed between groups (p < 0.001).

**Table 1:** Baseline characteristics of COPD participants.

Characteristic	Overall N = 7,700,327	Non-anemia N = 6,894,777	Anemia N = 805,550	*p*^a^
Unweighted numbers, n	1338	1134	204	
Age, years, mean (SE)	61.85 (0.34)	61.36 (0.35)	66.10 (1.12)	<0.001
Sex, n (unweighted) (%)				0.94
Male	759 (50.91%)	636 (50.87%)	123 (51.30%)	
Female	579 (49.09%)	498 (49.13%)	81 (48.70%)	
Race, n (unweighted) (%)				<0.001
Mexican American	875 (82.61%)	773 (84.46%)	102 (66.72%)	
Non-Hispanic Black	221 (6.44%)	158 (5.27%)	63 (16.39%)	
Non-Hispanic White	67 (1.63%)	55 (1.49%)	12 (2.77%)	
Other Hispanic	87 (2.27%)	78 (2.28%)	9 (2.16%)	
Other-Including Multi-Racial	88 (7.06%)	70 (6.49%)	18 (11.95%)	
Marry, n (unweighted) (%)				0.009
Married/Living with partner	744 (64.17%)	650 (65.54%)	94 (52.42%)	
Never married/Other	594 (35.83%)	484 (34.46%)	110 (47.58%)	
Education 1, n (unweighted) (%)				0.008
Less than high school	396 (20.71%)	327 (19.42%)	69 (31.73%)	
High school or equivalent	342 (26.22%)	295 (26.86%)	47 (20.77%)	
Above high school	600 (53.07%)	512 (53.72%)	88 (47.49%)	
Smoking status, n (unweighted) (%)				0.010
Never	207 (17.12%)	172 (16.40%)	35 (23.31%)	
Former	652 (47.71%)	539 (47.00%)	113 (53.82%)	
Current	479 (35.16%)	423 (36.60%)	56 (22.87%)	
Drinking status, n (unweighted) (%)				0.001
Never	91 (5.59%)	75 (5.16%)	16 (9.19%)	
Former	460 (30.14%)	365 (28.66%)	95 (42.76%)	
Now	787 (64.28%)	694 (66.17%)	93 (48.04%)	
PIR, n (unweighted) (%)				<0.001
≤1.3 (low income)	486 (24.12%)	403 (23.09%)	83 (32.90%)	
1.3∼3.5 (medium income)	506 (38.20%)	427 (37.57%)	79 (43.66%)	
>3.5 (high income)	346 (37.68%)	304 (39.34%)	42 (23.44%)	
Physical activity, min/week, mean (SE)	632.82 (46.31)	671.24 (52.33)	304.02 (51.96)	<0.001
Obesity, n (unweighted) (%)				0.060
BMI*<*30 kg/m^2^	799 (59.19%)	686 (60.13%)	113 (51.16%)	
BMI≥30 kg/m^2^	539 (40.81%)	448 (39.87%)	91 (48.84%)	
PLT, 10^9^/L, mean (SE)	252.99 (2.55)	252.34 (2.66)	258.53 (8.73)	0.72
Lymphocyte,10^9^/L, mean (SE)	2.10 (0.03)	2.11 (0.03)	1.97 (0.17)	<0.001
Neutrophils,10^9^/L, mean (SE)	4.67 (0.06)	4.67 (0.06)	4.64 (0.19)	0.55
Hemoglobin, g/dL, mean (SE)	14.27 (0.07)	14.59 (0.07)	11.53 (0.08)	<0.001
CVD, n (unweighted) (%)	431 (28.04%)	329 (25.90%)	102 (46.32%)	<0.001
Hypertension, n (unweighted) (%)	877 (60.04%)	708 (57.58%)	169 (81.15%)	<0.001
Diabetes, n (unweighted) (%)	422 (25.86%)	322 (23.82%)	100 (43.37%)	<0.001
CKD, n (unweighted) (%)	418 (25.03%)	306 (21.86%)	112 (52.23%)	<0.001
Asthma, n (unweighted) (%)	603 (44.05%)	496 (43.01%)	107 (52.95%)	0.059
Cancer, n (unweighted) (%)	279 (24.21%)	236 (24.01%)	43 (25.97%)	0.68
All-cause mortality, n (unweighted) (%)	436 (25.40%)	327 (22.14%)	109 (53.29%)	<0.001
CVD mortality, n (unweighted) (%)	98 (5.66%)	62 (4.31%)	36 (17.16%)	<0.001
Follow up, months, mean (SE)	88.35 (2.09)	91.34 (2.21)	62.79 (3.74)	<0.001

Note.

**N** represents weighted counts to reflect the population distribution, while **n** represents unweighted counts from the actual sample size.

Data on continuous variables were given as means and standard errors; data on categorical variables were presented as unweighted numbers and weighted percentage frequencies (%). all means and SEs for continuous variables and percentages for categorical variables were weighted.

Abbreviations: COPD, chronic obstructive pulmonary disease; PIR, Poverty Income Ratio; BMI, body mass index; CVD, chronic vascular disease, Includes congestive heart failure, coronary heart disease, angina, heart attack, and stroke; CKD, chronic kidney disease.

a Wilcoxon rank-sum test for complex survey samples; chi-squared test with Rao & Scott's second-order correction.

### 3.2 Association of anemia status and hemoglobin levels with all-cause and CVD mortality

Table 2 showed the associations between anemia status and hemoglobin levels with mortality in COPD participants. Anemia was significantly associated with all-cause mortality in COPD patients. In the fully adjusted model, anemia was associated with a 2.32-fold increased risk of all-cause mortality (HR = 2.32, 95%CI: 1.85-2.92, p < 0.001). Each 1 g/dL increase in hemoglobin level was associated with a 17% reduction in all-cause mortality risk (HR = 0.83, 95%CI: 0.76-0.91, p < 0.001).

**Table 2:** Association between anemia and mortality in COPD participants.

Variables	Crude Model		Model 1		Model 2		Model 3	
	HR (95%CI)	*p*	HR (95%CI)	*p*	HR (95%CI)	*p*	HR (95%CI)	*p*
**All-cause mortality**								
**Anemia**								
No	1.00 (Reference)		1.00 (Reference)		1.00 (Reference)		1.00 (Reference)	
Yes	3.73 (2.80, 4.98)	<0.001	2.89 (2.28, 3.67)	<0.001	2.65 (2.10, 3.33)	<0.001	2.32 (1.85, 2.92)	<0.001
**Hemoglobin, g/dL**	0.80 (0.74, 0.87)	<0.001	0.80 (0.73, 0.88)	<0.001	0.80 (0.74, 0.88)	<0.001	0.83 (0.76, 0.91)	<0.001
**CVD mortality**								
**Anemia**								
No	1.00 (Reference)		1.00 (Reference)		1.00 (Reference)		1.00 (Reference)	
Yes	6.23 (3.60, 10.78)	<0.001	5.51 (3.04, 10.00)	<0.001	4.94 (2.81, 8.72)	<0.001	3.75 (2.06, 6.83)	<0.001
**Hemoglobin, g/dL**	0.82 (0.68, 0.98)	0.031	0.73 (0.60, 0.89)	0.002	0.73 (0.61, 0.88)	<0.001	0.78 (0.66, 0.92)	0.003

**Crude model:** Not adjusted.

**Model 1:** Adjusted for age, sex, race, marital status, education level, smoking status, drinking status.

**Model 2:** Adjusted for age, sex, race, marital status, education level, smoking status, drinking status, physical activity time, PIR, BMI, lymphocyte cell counts, PLT, neutrophils cell counts.

**Model3:** Adjusted for age, sex, race, marital status, education level, smoking status, drinking status, physical activity time, PIR, BMI, lymphocyte cell counts, PLT, neutrophils cell counts, hypertension, diabetes, CVD, CKD, asthma, Cancer.

Abbreviations: COPD, chronic obstructive pulmonary disease; HR, hazard ratio; CI, confidence interval; PIR, Poverty Income Ratio; BMI, body mass index; CVD, chronic vascular disease; CKD, chronic kidney disease.

The association between anemia and CVD mortality was stronger. After full adjustment, anemia was associated with a 3.75-fold increased risk of CVD mortality (HR = 3.75, 95%CI: 2.06-6.83, p < 0.001). Each 1 g/dL increase in hemoglobin reduced CVD mortality risk by 22% (HR = 0.78, 95%CI: 0.66-0.92, p = 0.003).

### 3.3 Nonlinear relationship between hemoglobin levels with all-cause and CVD mortality

**Fig. 2:**
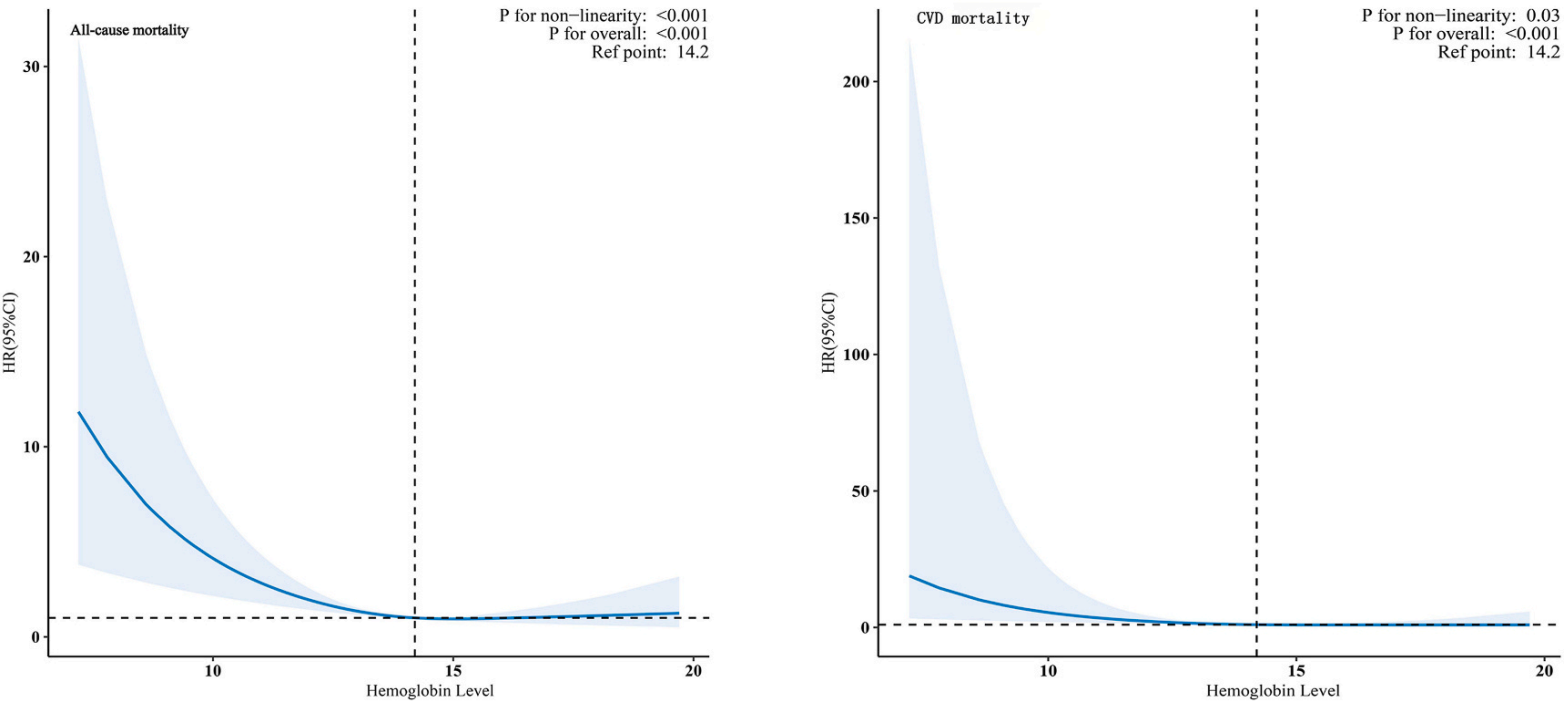
The nonlinear associations between hemoglobin level and mortality with COPD. Adjusted for age, sex, race, marital status, education level, smoking status, drinking status, physical activity time, PIR, BMI, lymphocyte cell counts, PLT, neutrophils cell counts, hypertension, diabetes, CVD, CKD, asthma, Cancer. **Abbreviation:** COPD, chronic obstructive pulmonary disease; PIR, poverty income ratio; BMI, body mass index; CVD, chronic vascular disease; CKD, chronic kidney disease.

In the fully adjusted model, restricted cubic spline analysis identified nonlinear dose-response relationships between hemoglobin levels and both all-cause and cardiovascular mortality (Fig. 2). Using a two-piecewise Cox regression model, hemoglobin thresholds were determined as 12.5 g/dL for all-cause mortality and 13.0 g/dL for cardiovascular mortality (Table 3). For all-cause mortality, each 1-g/dL increase in hemoglobin below 12.5 g/dL was associated with a 43% lower risk (HR, 0.57; 95% CI, 0.45–0.73; P < 0.001). No significant association was observed above this threshold (HR, 0.97; 95% CI, 0.86–1.09; P = 0.62).

**Table 3:** Threshold effects analysis of the association between hemoglobin level and mortality with COPD.

Threshold of hemoglobin level	HR (95%CI)	*p*
**All-cause mortality**		
hemoglobin level<12.5 g/dL	0.57(0.45, 0.73)	<0.001
hemoglobin level≥12.5 g/dL	0.97(0.86, 1.09)	0.62
**CVD mortality**		
hemoglobin level<13.0 g/dL	0.64(0.42, 0.97)	0.03
hemoglobin level≥13.0 g/dL	1.10(0.85, 1.42)	0.47

**Note:** Adjusted for age, sex, race, marital status, education level, smoking status, drinking status, physical activity time, PIR, BMI, lymphocyte cell counts, PLT, neutrophils cell counts, hypertension, diabetes, CVD, CKD, asthma, Cancer.

**Abbreviations:** COPD, chronic obstructive pulmonary disease; HR, hazard ratio; CI, confidence interval; PIR, Poverty Income Ratio; BMI, body mass index; CVD, chronic vascular disease; CKD, chronic kidney disease.

For cardiovascular mortality, each 1-g/dL increase in hemoglobin below 13.0 g/dL was associated with a 36% lower risk (HR, 0.64; 95% CI, 0.42–0.97; P = 0.03). Above this threshold, the association was not significant (HR, 1.10; 95% CI, 0.85–1.42; P = 0.47).

### 3.4 Survival analysis

**Fig. 3:**
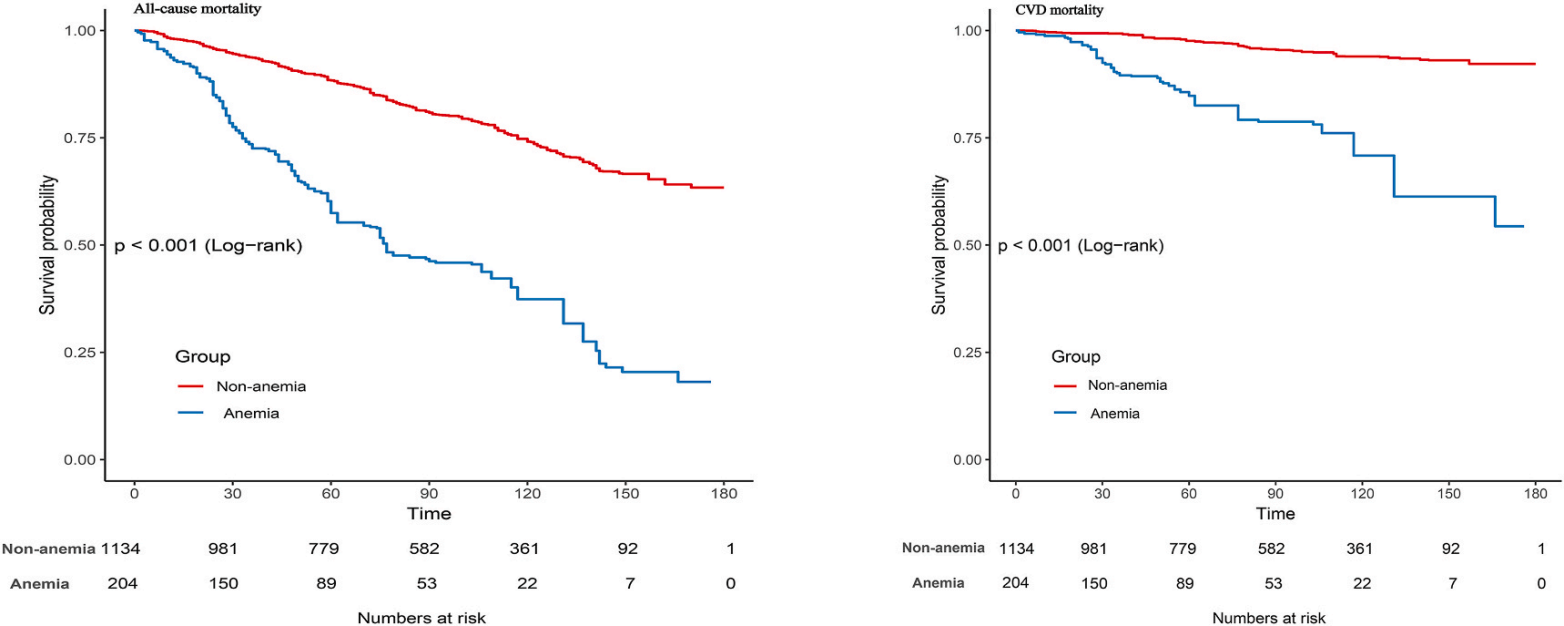
Kaplan-Meier survival curve for all-cause and CVD mortality of COPD individuals. **Abbreviation:** COPD, chronic obstructive pulmonary disease; CVD, chronic vascular disease; CKD, chronic kidney disease.

During a median follow-up period of 92 months, 436 al l-cause deaths occurred, including 98 CVD deaths. Kaplan–Meier curves demonstrated significantly lower survival among participants with anemia compared to those without (log-rank P < 0.001) (Fig. 3).

### 3.5 Subgroup and sensitivity analyses

**Fig. 4:**
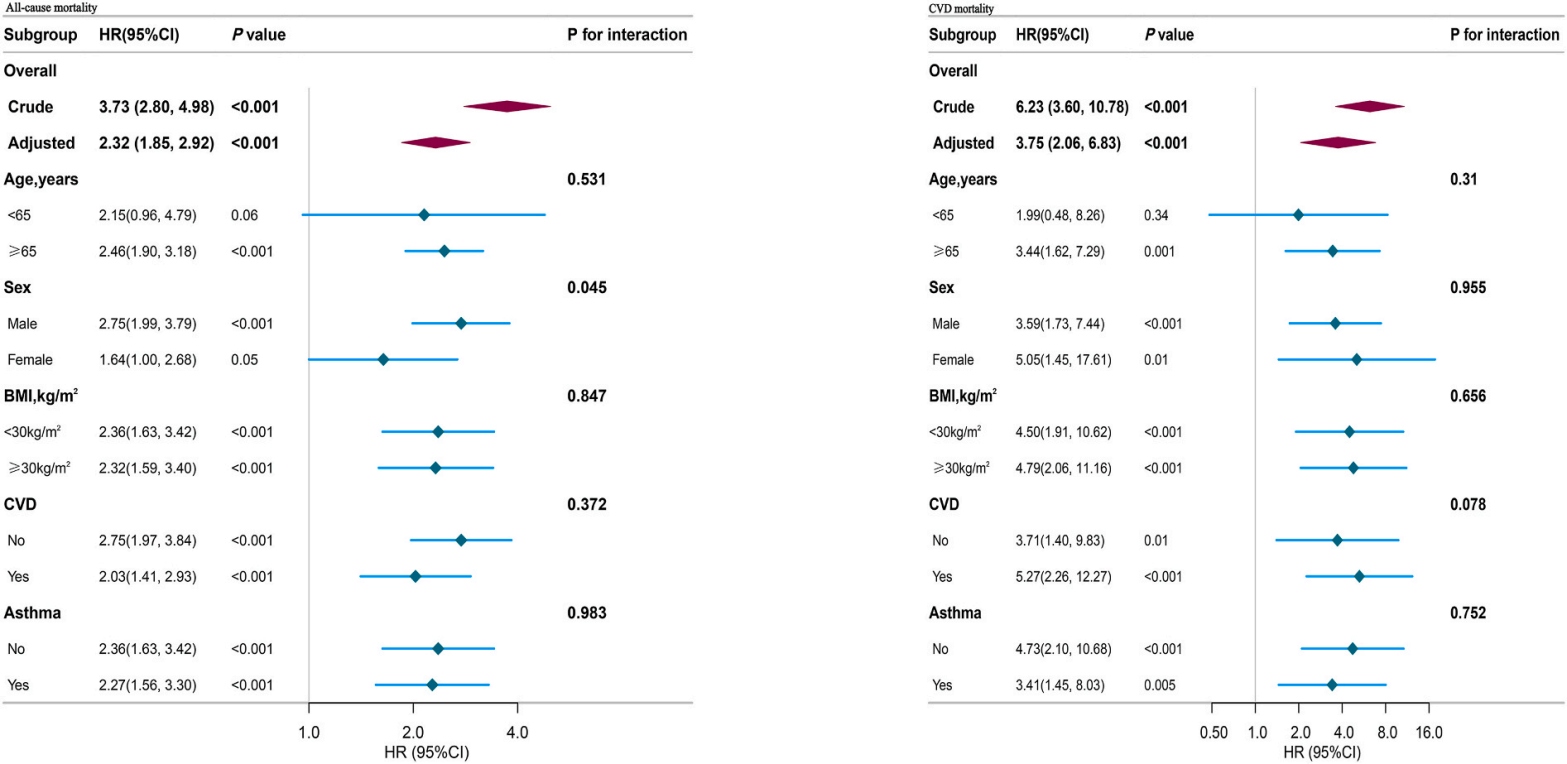
Subgroup analysis of the association of between hemoglobin level and mortality with COPD. Adjusted for age, sex, race, marital status, education level, smoking status, drinking status, physical activity time, PIR, BMI, lymphocyte cell counts, PLT, neutrophils cell counts, hypertension, diabetes, CVD, CKD, asthma, and cancer except subgroup itself. **Abbreviation:** COPD, chronic obstructive pulmonary disease; HR, hazard ratio; CI, confidence interval; PIR, Poverty Income Ratio; BMI, body mass index; CVD, chronic vascular disease; CKD, chronic kidney disease.

Subgroup analyses are presented in Fig. 4. The association between anemia and all-cause mortality was significantly stronger in males (*p* for interaction = 0.045), with no significant effect modification by age, BMI, CVD, or asthma status. For CVD mortality, the association was consistent across all subgroups with no significant interactions.

Sensitivity analyses confirmed the robustness of the main findings (Table 4). After excluding deaths within 2 years of follow-up, anemia remained associated with increased risks of all-cause mortality (HR, 2.09; 95% CI, 1.59–2.76; P < 0.001) and cardiovascular mortality (HR, 3.54; 95% CI, 2.06–6.10; P < 0.001). Multiple imputation and unweighted analyses yielded consistent results.

**Table 4:** Sensitive analysis.

Variables	Crude Model			Model 1			Model 2			Model 3	
	HR (95%CI)	*p*		HR (95%CI)	*p*		HR (95%CI)	*p*		HR (95%CI)	*p*
**Excluded 2 years death**											
**All-cause mortality**											
Anemia(yes/no)	2.81 (2.19∼3.62)	<0.001		2.47 (1.89∼3.22)	<0.001		2.36 (1.8∼3.09)	<0.001		2.09 (1.59∼2.76)	<0.001
**CVD mortality**											
Anemia(yes/no)	4.81 (2.99∼7.74)	<0.001		4.36 (2.6∼7.33)	<0.001		4.45 (2.62∼7.56)	<0.001		3.54 (2.06∼6.1)	<0.001
**Multiple imputation**											
**All-cause mortality**											
Anemia(yes/no)	2.27 (1.73∼2.98)	<0.001		1.91 (1.39∼2.63)	<0.001		1.78 (1.29∼2.46)	0.001		1.57 (1.13∼2.19)	0.008
**CVD mortality**											
Anemia(yes/no)	3.02 (2.03∼4.5)	<0.001		2.5 (1.6∼3.89)	<0.001		2.58 (1.58∼4.23)	<0.001		1.85 (1.15∼2.95)	0.011
**Unweighted results**											
**All-cause mortality**											
Anemia(yes/no)	2.82 (2.26∼3.51)	<0.001		2.34 (1.85∼2.96)	<0.001		2.24 (1.77∼2.84)	<0.001		1.96 (1.54∼2.5)	<0.001
**CVD mortality**											
Anemia(yes/no)	4.92 (3.25∼7.46)	<0.001		4.17 (2.65∼6.57)	<0.001		3.97 (2.49∼6.31)	<0.001		3.03 (1.88∼4.89)	<0.001

**Crude model:** Not adjusted.

**Model 1:** Adjusted for age, sex, race, marital status, education level, smoking status, drinking status.

**Model 2:** Adjusted for age, sex, race, marital status, education level, smoking status, drinking status, physical activity time, PIR, BMI, lymphocyte cell counts, PLT, neutrophils cell counts.

**Model3:** Adjusted for age, sex, race, marital status, education level, smoking status, drinking status, physical activity time, PIR, BMI, lymphocyte cell counts, PLT, neutrophils cell counts, hypertension, diabetes, CVD, CKD, asthma, Cancer.

Abbreviations: COPD, chronic obstructive pulmonary disease; HR, hazard ratio; CI, confidence interval; PIR, Poverty Income Ratio; BMI, body mass index; CVD, chronic vascular disease; CKD, chronic kidney disease.

Propensity score analyses confirmed robust associations between anemia and mortality across all methodological approaches (Table 5). After propensity score matching, anemia remained associated with 1.87-fold increased all-cause mortality (95%CI: 1.38-2.52, p < 0.001) and 2.63-fold increased CVD mortality (95%CI: 1.46-4.75, p = 0.001).

**Table 5:** Association of the anemia with mortality in propensity score.

Models	All-cause mortality		CVD mortality	
	HR (95%CI)	*P*	HR (95%CI)	*P*
Unmatched. crude	2.82 (2.26∼3.51)	<0.001	4.92 (3.25∼7.46)	<0.001
Multivariable adjusted a	1.96 (1.54∼2.5)	<0.001	3.03 (1.88∼4.89)	<0.001
Propensity Score. adjusted b	1.87 (1.46∼2.38)	<0.001	2.78 (1.74∼4.46)	<0.001
Propensity Score. Matched c	1.87 (1.38∼2.52)	<0.001	2.63 (1.46∼4.75)	0.001
Weighted. IPTW d	1.78 (1.42∼2.24)	0.004	2.24 (1.37∼3.65)	0.006
Weighted. SMRW e	1.83 (1.48∼2.25)	<0.001	3.11 (2.1∼4.61)	<0.001
Weighted.PA f	1.79 (1.33∼2.4)	<0.001	2.96 (1.61∼5.43)	<0.001
Weighted. Ow g	1.82 (1.29∼2.59)	<0.001	2.8 (1.36∼5.8)	<0.001

Note.

a Shown is the hazard ratio from the multivariable Cox proportional hazards model, adjusted for all covariates.

b Shown is the hazard ratio from a multivariable Cox proportional-hazards model with the same strata and covariates, with additional adjustment for the propensity score.

c Shown is the hazard ratio from a multivariable Cox proportional-hazards model with the same strata and covariates matching according to the propensity score.

d Shown is the primary analysis with a hazard ratio from the multivariable Cox proportional-hazards model with the same strata and covariates with inverse probability weighting according to the propensity score.

e Shown is the primary analysis with a hazard ratio from the multivariable Cox proportional-hazards model with the same strata and covariates with the standardized mortality ratio weighting according to the propensity score.

f Shown is the primary analysis with a hazard ratio from the multivariable Cox proportional-hazards model with the same strata and covariates with pairwise algorithmic according to the propensity score.

g Shown is the primary analysis with a hazard ratio from the multivariable Cox proportional-hazards model with the same strata and covariates with overlap weight according to the propensity score.

## 4 Discussion

In this nationally representative cohort study, the results demonstrated that anemia was significantly associated with all-cause (HR = 2.32) and cardiovascular mortality (HR = 3.75). Nonlinear dose-response relationships between hemoglobin levels and mortality were identified, with threshold effects at 12.5 g/dL for all-cause mortality and 13.0 g/dL for cardiovascular mortality. These findings were robust across extensive sensitivity analyses including propensity score-based methods and remained consistent across most demographic and clinical subgroups. While these findings identify hemoglobin as a prognostic indicator, the observational design does not establish causality, and these thresholds should be regarded as hypothesis-generating risk markers rather than therapeutic targets.

Our findings align with and extend prior research on anemia and adverse outcomes in COPD. Previous studies have indicated that anemia is associated with increased mortality following COPD exacerbations [32], and longitudinal cohorts have demonstrated that anemia is linked to frequent exacerbations, quality of life decline, and pulmonary function deterioration in COPD [33]. A nationwide inpatient database analysis in the United States reported that anemia was associated with significant resource utilization, healthcare costs, and mortality in patients hospitalized for acute exacerbations [18]. Additionally, a Korean nationwide cohort study reported a hazard ratio of 1.31 for anemia-associated mortality [15]. A separate study found that lower hemoglobin at admission was associated with higher long-term mortality after discharge in COPD patients [34]. Above studies were limited to all-cause outcomes without discrimination of specific causes of death. Our findings extend the existing literature by providing a comprehensive assessment of both all-cause and cardiovascular mortality. The hazard ratio for cardiovascular mortality (HR = 3.75, 95% CI: 2.06–6.83) appeared numerically larger than that for all-cause mortality (HR = 2.32, 95% CI: 1.85–2.92). This analytical restriction is clinically significant given that cardiovascular disease represents the leading cause of mortality in COPD populations, and anemia may exert differential pathophysiological effects on cardiac-specific versus non-cardiac mortality through distinct mechanisms including hemodynamic stress, exacerbation of tissue hypoxemia, and promotion of atherothrombotic events [35,36]. Several pathophysiological mechanisms may explain this pattern, although we did not perform a formal statistical comparison between these two hazard ratios and therefore cannot conclude that the difference is statistically significant.

A novel finding of this study is the identification of distinct hemoglobin thresholds for mortality risk. The threshold of 12.5 g/dL for all-cause mortality and 13.0 g/dL for cardiovascular mortality, consistent with recent evidence suggesting L-shaped or threshold associations rather than linear patterns [37]. Notably, this comparable recent analysis identified a higher inflection point of 14.2 g/dL for all-cause mortality. This discrepancy likely reflects key methodological differences: this weighted analysis of 1338 participants ensured national representativeness of the U.S. COPD population and employed comprehensive diagnostic criteria incorporating spirometry, physician diagnosis, and clinical indicators, whereas the prior study used unweighted data from limited cycles with potentially less rigorous case ascertainment. Additionally, our use of survey-weighted restricted cubic splines appropriately accounted for the complex sampling design, yielding more precise population-level estimates (12.5–13.0 g/dL) that align closely with WHO anemia definitions (12–13 g/dL), rather than the higher, less clinically actionable threshold of 14.2 g/dL. Importantly, these thresholds should not be interpreted as diagnostic or therapeutic cutoffs, as the study did not evaluate causes of anemia or the effects of treatment. Rather, these values represent prognostic risk markers that may help identify COPD patients who could benefit from closer cardiovascular surveillance and more intensive risk factor modification. Future prospective studies are needed to validate these thresholds and to determine whether hemoglobin-guided risk stratification can improve clinical outcomes.

The robustness of our findings is supported by comprehensive sensitivity analyses, including exclusion of early deaths, multiple imputation for missing covariates, unweighted regression, and seven distinct propensity score approaches (matching, adjustment, IPTW, SMRW, PA, and OW), all yielding consistent effect estimates. Regarding subgroup analyses, a nominally significant interaction between anemia and sex was observed for all-cause mortality (p for interaction = 0.045). Given the borderline statistical significance, the multiple subgroup comparisons performed, and the exploratory nature of these analyses, this finding should be interpreted with considerable caution and may represent a Type I error. Whether it reflects genuine biological differences (e.g., differential baseline hematologic profiles, sex-specific cardiovascular reserve, or hormonal modulation of erythropoiesis) requires independent replication.

This study utilized NHANES data with rigorous standardized protocols and complex sampling designs ensuring national representativeness. The extensive sensitivity analyses, particularly seven different propensity score approaches, provide robust evidence against confounding by indication or selection bias. However, several limitations should be acknowledged. First, the observational design precludes definitive causal inference; residual confounding from unmeasured variables (including inflammatory burden markers such as C-reactive protein, COPD severity indices, and treatment-related factors including long-term oxygen therapy, diuretics, renin-angiotensin system blockers, and SGLT2 inhibitors, all of which may influence both hemoglobin levels and cardiovascular risk) cannot be entirely excluded. Second, COPD diagnosis was based on a composite of spirometry, physician diagnosis, and medication use rather than spirometry alone, which may introduce misclassification bias; however, this approach is consistent with prior NHANES-based research and enhances sensitivity in population-based settings where spirometry data may be incomplete [[21], [22], [23]]. Third, hemoglobin was measured at a single baseline time point, which may not capture intra-individual variability, longitudinal changes, or the influence of acute illness, hydration status, or COPD exacerbation on hemoglobin levels. Fourth, this study was not designed to determine whether correction of anemia improves outcomes; the clinical implications of these findings are limited to risk stratification and should not be interpreted as evidence supporting specific therapeutic interventions. Finally, our findings are derived from the U.S. population and may not be directly generalizable to other geographic or healthcare settings.

These findings have several potential clinical implications. First, hemoglobin assessment—a routinely available, inexpensive laboratory parameter—may serve as an adjunctive tool for cardiovascular risk stratification in patients with COPD, particularly those with hemoglobin levels between 12.0 and 14.0 g/dL where standard anemia criteria may not flag risk. Clinicians caring for COPD patients with hemoglobin below 13.0 g/dL might consider more intensive cardiovascular evaluation, including assessment for concurrent heart failure, coronary artery disease, and arrhythmia. Second, hemoglobin values below these thresholds could prompt evaluation for potentially reversible causes of anemia, including iron deficiency, vitamin B12/folate insufficiency, and CKD-related erythropoietin deficiency. Importantly, while these observations generate hypotheses regarding the potential benefit of anemia correction, randomized controlled trials are necessary to establish whether targeted anemia treatment—through iron supplementation, erythropoiesis-stimulating agents, or management of underlying etiologies—can reduce mortality in patients with COPD. Future prospective studies with repeated hemoglobin measurements are also needed to clarify the temporal dynamics of anemia and mortality risk and to validate the hemoglobin thresholds identified in the present analysis.

## 5 Conclusion

In conclusion, in this large, nationally representative cohort of U.S. adults with COPD, anemia and lower hemoglobin levels were independently associated with significantly increased risks of all-cause and cardiovascular mortality. We identified hemoglobin thresholds of 12.5 g/dL and 13.0 g/dL for all-cause and cardiovascular mortality, respectively. These findings highlight the prognostic importance of hemoglobin assessment in COPD and suggest that hemoglobin levels below 13.0 g/dL may warrant heightened cardiovascular surveillance in this population. Given the observational nature of this study, randomized controlled trials are necessary to determine whether targeted intervention for anemia can improve survival in patients with COPD.

## Funding statement

None.

## CRediT authorship contribution statement

**Yan Wang:** Formal analysis, Investigation, Methodology, Resources, Software, Validation, Writing – original draft. **Yushan Shi:** Conceptualization, Data curation, Formal analysis, Investigation, Methodology, Project administration, Resources, Validation, Visualization, Writing – review & editing.

## Declaration of competing interest

The authors declare that they have no known competing financial interests or personal relationships that could have appeared to influence the work reported in this paper.
